# Live imaging of H3K9 acetylation in plant cells

**DOI:** 10.1038/srep45894

**Published:** 2017-04-18

**Authors:** Kazuki Kurita, Takuya Sakamoto, Noriyoshi Yagi, Yuki Sakamoto, Akihiro Ito, Norikazu Nishino, Kaori Sako, Minoru Yoshida, Hiroshi Kimura, Motoaki Seki, Sachihiro Matsunaga

**Affiliations:** 1 Department of Applied Biological Science, Faculty of Science and Technology, Tokyo University of Science, 2641 Yamazaki, Noda, Chiba 278-8510, Japan; 2 Imaging Frontier Center, Organization for Research Advancement, Tokyo University of Science, 2641 Yamazaki, Noda, Chiba 278-8510, Japan; 3 Chemical Genomics Research Group, RIKEN Center for Sustainable Resource Science, 2-1 Hirosawa, Wako, Saitama, 351-0198, Japan; 4 Graduate School of Life Science and Systems Engineering, Kyushu Institute of Technology, Kitakyushu, 808-0196, Japan; 5 Plant Genomic Network Research Team, RIKEN Center for Sustainable Resource Science, 1-7-22 Suehiro-cho, Tsurumi-ku, Yokohama, Kanagawa, 230-0045, Japan; 6 Graduate School of Bioscience and Biotechnology, Tokyo Institute of Technology, 4259 Nagatsuta, Midori-ku, Yokohama, 226-8501, Japan

## Abstract

Proper regulation of histone acetylation is important in development and cellular responses to environmental stimuli. However, the dynamics of histone acetylation at the single-cell level remains poorly understood. Here we established a transgenic plant cell line to track histone H3 lysine 9 acetylation (H3K9ac) with a modification-specific intracellular antibody (mintbody). The H3K9ac-specific mintbody fused to the enhanced green fluorescent protein (H3K9ac-mintbody-GFP) was introduced into tobacco BY-2 cells. We successfully demonstrated that H3K9ac-mintbody-GFP interacted with H3K9ac *in vivo*. The ratio of nuclear/cytoplasmic H3K9ac-mintbody-GFP detected in quantitative analysis reflected the endogenous H3K9ac levels. Under chemically induced hyperacetylation conditions with histone deacetylase inhibitors including trichostatin A, Ky-2 and Ky-14, significant enhancement of H3K9ac was detected by H3K9ac-mintbody-GFP dependent on the strength of inhibitors. Conversely, treatment with a histone acetyltransferase inhibitor, C646 caused a reduction in the nuclear to cytoplasmic ratio of H3K9ac-mintbody-GFP. Using this system, we assessed the environmental responses of H3K9ac and found that cold and salt stresses enhanced H3K9ac in tobacco BY-2 cells. In addition, a combination of H3K9ac-mintbody-GFP with 5-ethynyl-2′-deoxyuridine labelling confirmed that H3K9ac level is constant during interphase.

Epigenetic regulation based on histone modification is essential for numerous biological processes in eukaryotic organisms^1^,^2^. The dynamics of histone modification have been mainly analysed by chromatin immunoprecipitation (ChIP) and immunofluorescence with antibodies, which specifically recognize modified amino acids in histones. Because cell populations or whole tissues are generally used as experimental materials for ChIP, studying the dynamics of histone modification at the single-cell level is difficult^3^. Time-course analyses of histone modification in the same cell cannot be performed using immunofluorescence because the preparation needs the fixation of cells^4^. Histone modification is changeable at the single-cell level in response to environmental stresses and during development and differentiation^5^,^6^. Thus, to enable us to four-dimensionally detect histone modification at the single-cell level, a live cell imaging method is required. One solution is a Forster/fluorescence resonance energy transfer (FRET)-based biosensor to detect histone modification^7^. Intermolecular FRET sensors that can detect acetylation of histone H3 at K9 or K14 and H4 at K12 have been developed^8^,^9^. Although FRET sensors are available for chemical screening of cultured animal cells, analyses at the single-cell level in multicellular tissues and organs are practically difficult^10^.

Another solution is to use a modification-specific intracellular antibody (mintbody)^6^,^11^. A mintbody is a genetically encoded exogenous protein with a single-chain variable fragment (scFv) fused to a fluorescent protein. H3K9ac-mintbody is expressed through a vector with the scFV coding sequence from mouse hybridoma cells that produces a monoclonal antibody against histone H3 Lys9 acetylation (H3K9ac). The acetylation level can be evaluated with the mintbody by the ratio of nuclear to cytoplasmic intensity of the fluorescent protein fused with the scFv. An increase in H3K9ac was detected as an increase in the ratio of nuclear to cytoplasmic intensity when a histone deacetylase inhibitor was added to hTERT-RPE1 cells expressing H3K9ac-mintbody^11^. Transgenic animals stably expressing H3K9ac-mintbody have been constructed in fruit fly (*Drosophila melanogaster*), zebrafish (*Danio rerio*) and *Xenopus laevis*^11^,^12^,^13^. An increase of H3K9ac could be monitored in specific regions during embryogenesis and organ development of these transgenic animals. Recently, yeast (*Schizosaccharomyces pombe*), nematode (*Caenorhabditis elegans*), mouse and mammalian cells expressing H4K20me1-mintbody were constructed and oscillation of H4K20me1 was detected during the cell cycle^14^.

In plant epigenetic regulation, H3K9ac is a euchromatic marker that is preferentially localized at gene-rich regions^15^. Thus, H3K9ac is closely correlated with transcriptional activation during plant development and differentiation processes including flowering^16^ and leaf aging^17^. Moreover, dynamic changes of H3K9ac have been found in response to environmental stimuli or stresses^18^. H3K9ac changed dynamically during de-etiolation in response to light^19^ and after irradiation with UV light^20^, X-rays^21^ and gamma-rays^22^. Moreover, H3K9ac increased in drought-responsive genes under drought stress and decreased during rehydration^23^,^24^. However, all detection of H3K9ac in these analyses was performed by ChIP, mass-spectrometry and immunofluorescence. Thus, to analyse H3K9ac at the single-cell level in plants, we attempted to track H3K9ac *in vivo* using a mintbody. Although problems expressing the mouse scFv properly in plant cells were concerned, we report here the detection of H3K9ac using the mintbody in living plant cells.

## Results

### Introduction of a mintbody against acetylated H3K9 into tobacco BY-2 cells

**Figure 1:**
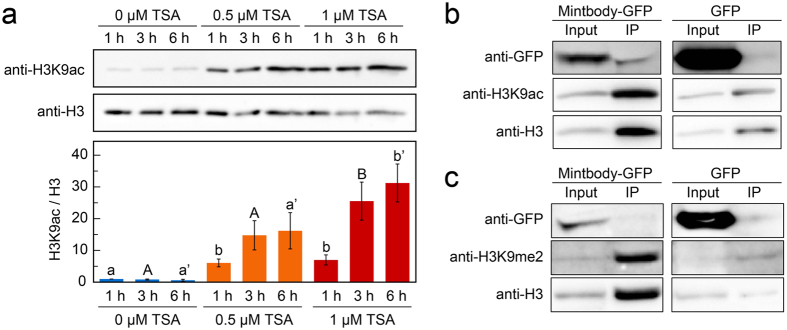
Interaction of H3K9ac-mintbody-GFP with acetylated H3K9 in tobacco BY-2 cells. (**a**) H3K9ac levels in response to TSA treatments in BY-2 cells. The upper panel shows immunoblotting of H3K9ac and H3. The lower panel shows the quantification of H3K9ac levels calculated from the intensity of each band. H3K9ac levels were normalized with H3 levels. The values represent means ± S.D.. Distinct letters on the bars indicate significant difference at each treatment time (n = 3, Tukey’s multiple range test: *p* < 0.05). For the full-length western blots see Supplementary Fig. S4a. (**b**) Analysis of co-immunoprecipitation of endogenous H3K9ac with H3K9ac-mintbody-GFP in H3K9ac-mintbody-GFP expressing tobacco BY-2 cells treated with 1 μM TSA for 3 h. For the full-length western blots see Supplementary Fig. S4b. (**c**) Analysis of co-immunoprecipitation of endogenous H3K9me2 with H3K9ac-mintbody-GFP in H3K9ac-mintbody-GFP expressing tobacco BY-2 cells treated with 10 μM C646 for 3 h. For the full-length western blots see Supplementary Fig. S4c.

Because the H3K9ac-mintbody is derived from mouse hybridoma cells and plants do not have an acquired immune system like animals do, we first investigated whether heterologously expressed H3K9ac-mintbody worked as a functional antibody for H3K9ac in plant cells. To assess the reliability of the mintbody in plant cells, we used tobacco BY-2 cultured cells^25^. The histone acetylation level in the cells was upregulated using an inhibitor of histone deacetylases (HDACs), trichostatin A (TSA)^26^ (Fig. 1a). We found that a 3 h treatment of 1 μM TSA showed the highest H3K9ac level (Fig. 1a). In accordance with this result, we selected treatment conditions of 1 μM TSA for 3 h for subsequent analyses. Then, we generated a cell line expressing the H3K9ac-mintbody fused to the enhanced green fluorescent protein (GFP) under the control of the cauliflower mosaic virus (CaMV) 35S promoter and analysed the functionality of H3K9ac-mintbody-GFP by immunoprecipitation under histone hyperacetylation conditions. If the mintbody acts as a functional antibody, H3K9ac-mintbody-GFP should bind to H3K9ac, particularly in cells treated with TSA. Indeed, our immunoprecipitation analysis showed the interaction of H3K9ac-mintbody-GFP with H3K9ac (Fig. 1b), indicating that the mintbody worked as an antibody against H3K9ac in tobacco BY-2 cells. Surprisingly, we found that H3K9ac-mintbody-GFP was immunoprecipitated only using anti-mouse IgG antibody (Supplementary Fig. S1), strongly supporting that H3K9ac-mintbody-GFP was properly folded and functional as an antibody in plant cells.

Next, we evaluated the specificity of H3K9ac-mintbody-GFP to H3K9ac. We assumed that if H3K9ac-mintbody-GFP specifically bound to H3K9ac, it would not interact with other types of modifications at the same amino acid residue. To enrich the levels of H3K9me2 relative to H3K9ac, we treated cells with a histone acetyltransferase inhibitor, C646^27^. Treatment with 10 μM C646 for 3 h caused a reduction of H3K9ac levels in tobacco BY-2 cells (Supplementary Fig. S2a,b). Under this condition, H3K9ac-mintbody-GFP was not co-immunoprecipitated with an antibody against H3K9me2 (Fig. 1c), indicating that H3K9ac-mintbody-GFP was highly specific to H3K9ac in tobacco BY-2 cells.

**Figure 2:**
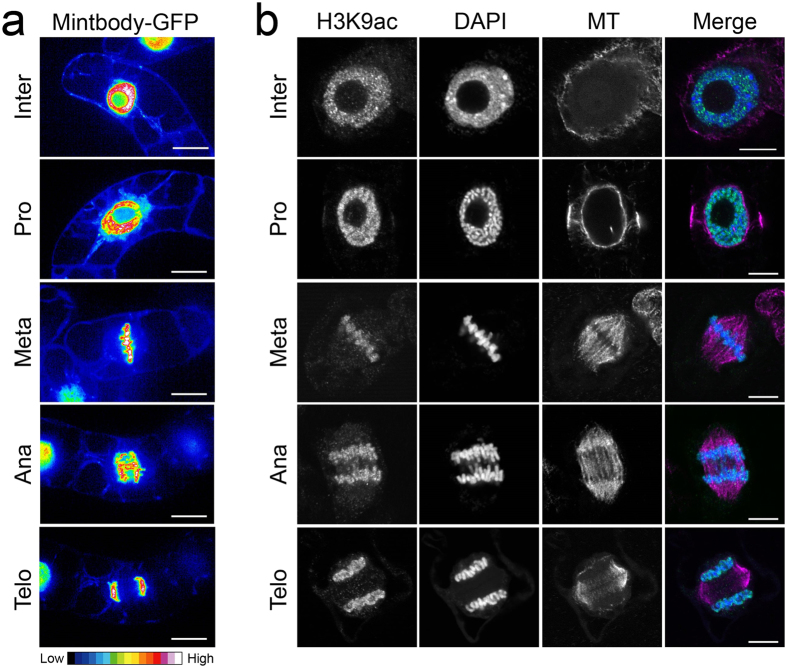
Dynamics of H3K9ac-mintbody-GFP and H3K9ac during mitosis in tobacco BY-2 cells. (**a**) Expression patterns of H3K9ac-mintbody-GFP in tobacco BY-2 cells. The intensity of GFP fluorescence is shown by a heat map. Bars, 50 μm. (**b**) Immunostaining of histone H3K9ac in tobacco BY-2 cells. In the merged image, acetylated H3K9, DNA, and microtubules are shown in green, blue, and magenta, respectively. Bars, 10 μm. Inter, interphase; Pro, prophase; Meta, metaphase; Ana, anaphase; Telo, telophase.

Next, we observed the localization of H3K9ac-mintbody-GFP in tobacco BY-2 cells under normal conditions using confocal microscopy. During interphase, H3K9ac-mintbody-GFP was localized in both the cytoplasm and nucleus, and a higher intensity of H3K9ac-mintbody-GFP was detected in the nucleus as previously observed in human cells^11^ (Fig. 2a). These localization patterns indicated that H3K9ac could be monitored in tobacco BY-2 cells by measuring the ratio of nuclear/cytoplasmic fluorescence intensity as is the case in animal cells^11^. We also detected a high intensity of H3K9ac-mintbody-GFP on mitotic chromosomes from prophase to telophase (Fig. 2a). Immunostaining analysis also demonstrated histone acetylation at H3K9 along mitotic chromosomes from prophase to telophase, as observed using H3K9ac-mintbody-GFP (Fig. 2b).

### Monitoring changes in acetylation levels of H3K9 in living tobacco BY-2 cells

**Figure 3:**
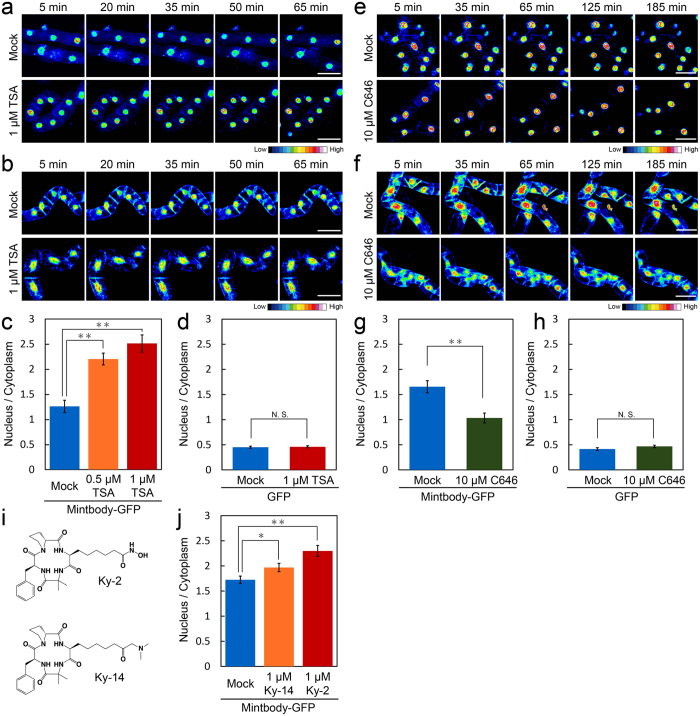
Monitoring of H3K9ac-mintbody-GFP in response to changes in acetylation status in tobacco BY-2 cells. (**a**,**b**) Time-lapse imaging of tobacco BY-2 cells expressing H3K9ac-mintbody-GFP (**a**) and GFP alone (**b**) in response to TSA treatment. The intensity of GFP fluorescence is shown by a heat map. The same cells were observed at the indicated time point. Bars, 100 μm. (**c**,**d**) Quantitative analysis of GFP levels in tobacco BY-2 cells expressing H3K9ac-mintbody-GFP (**c**) and GFP alone (**d**) treated with TSA for 3 h. The ratio of nuclear/cytoplasmic intensity of GFP is shown. The values represent means ± S.D. (n = 13–16, *t*-test; **p < 0.01, N.S., not significant). (**e**,**f**) Time-lapse imaging of tobacco BY-2 cells expressing H3K9ac-mintbody-GFP (**e**) and GFP alone (**f**) in response to C646 treatment. The intensity of GFP fluorescence is shown by a heat map. The same cells were observed at the indicated time point. Bars, 100 μm. (**g**,**h**) Quantitative analysis of GFP levels in tobacco BY-2 cells expressing H3K9ac-mintbody-GFP (**g**) and GFP alone (**h**) treated with C646 for 3 h. The ratio of nuclear/cytoplasmic intensity of GFP is shown. The values represent means ± S.D. (n = 14–18, N.S., not significant by *t*-test). (**i**) Chemical structures of synthesized HDAC inhibitors Ky-2 and Ky-14. (**j**) Quantitative analysis of GFP levels in tobacco BY-2 cells expressing H3K9ac-mintbody-GFP treated with Ky-2 and Ky-14 for 3 h. The ratio of nuclear/cytoplasmic intensity of GFP is shown. The values represent means ± S.D. (n = 34–38, *t*-test; *p < 0.05, **p < 0.01).

In cultured human cells, H3K9ac-mintbody-GFP is reversibly mobile between the cytoplasm and nucleus during interphase depending on the acetylation level of endogenous H3K9. When H3K9 is highly acetylated, H3K9ac-mintbody-GFP preferentially accumulates in nuclei in accordance with its decrease in the cytoplasm^11^. To evaluate whether this tendency was conserved in tobacco BY-2 cells, we conducted a quantitative analysis of the intensity of H3K9ac-mintbody-GFP under histone hyperacetylation conditions. Time-lapse imaging showed that when the cells were treated with TSA for 1 h, H3K9ac-mintbody-GFP became brighter in the nucleus and, conversely, darker in the cytoplasm (Fig. 3a). The quantified nuclear to cytoplasmic ratio of H3K9ac-mintbody-GFP indicated an increased level of endogenous H3K9 acetylation in a TSA dose-dependent manner, consistent with immunoblotting analyses (Figs 1a and 3c). In contrast, both time-lapse and quantitative analysis of cells overexpressing GFP under the CaMV 35S promoter revealed that the nuclear to cytoplasmic intensity ratio of GFP alone did not change in response to the TSA treatment (Fig. 3b,d). Conversely, treatment with the histone acetyltransferase inhibitor C646^27^, which decreased the levels of histone acetylation (Supplementary Fig. S2a,b), made H3K9ac-mintbody-GFP darker in the nucleus and brighter in the cytoplasm as time proceeded (Fig. 3e). Quantitative analysis revealed that the C646 treatment reduced the nuclear to cytoplasmic ratio of H3K9ac-mintbody-GFP (Fig. 3g). Similar to the TSA treatment, the dynamics of GFP alone did not change upon C646 treatment (Fig. 3f,h).

We also assessed the effects of other HDAC inhibitors. Ky-2^28^,^29^ is a cyclic tetrapeptide-based histone deacetylase inhibitor developed by RIKEN and the Kyushu Institute of Technology^29^. Ky-14, cyclo(-l-2-amino-8-oxo-9-dimethylaminononanoyl-aminoisobutylyl-l-phenylalanyl-d-prolyl-), was newly design-synthesized according to a previous report^30^. This cyclic tetrapeptide with dimethylaminomethylketone function showed satisfactory data in high-resolution mass spectrometry and NMR spectroscopy. Ky-14 showed moderate inhibitory activities against HDAC1 and HDAC4 compared with Ky-2 (Supplementary Table S1). Interestingly, consistent with the results of the *in vitro* assay, quantitative analysis showed a greater increase in the nuclear/cytoplasmic intensity of H3K9ac-mintbody-GFP in Ky-2 treated cells than in Ky-14 treated cells (Fig. 3f).

To further confirm the response of H3K9ac-mintbody-GFP, we evaluated changes in the amount of H3K9ac-mintbody-GFP in nuclei treated with TSA or C646 by immunoblotting analysis. The H3K9ac-mintbody-GFP levels in extracted nuclei changed in correlation with H3K9ac levels under TSA or C646 treatment conditions (Supplementary Fig. S2a–c). In contrast, neither the TSA nor the C646 treatment affected the total H3K9ac-mintbody-GFP levels in cells (Supplementary Fig. S2d,e). Therefore, these immunoblotting analyses indicated that the nuclear to cytoplasmic ratio of H3K9ac-mintbody-GFP was increased by the TSA treatment and decreased by the C646 treatment, which was consistent with the results of imaging analysis.

Taken together, these results confirmed that H3K9ac-mintbody-GFP was responsive to changes in the acetylation status of H3K9 in tobacco BY-2 cells. Therefore, measurement of the nuclear/cytoplasmic intensity of H3K9ac-mintbody-GFP enables quantitative monitoring of H3K9ac levels in living tobacco BY-2 cells.

### Monitoring the effects of environmental stresses on H3K9ac levels in living tobacco BY-2 cells

**Figure 4:**
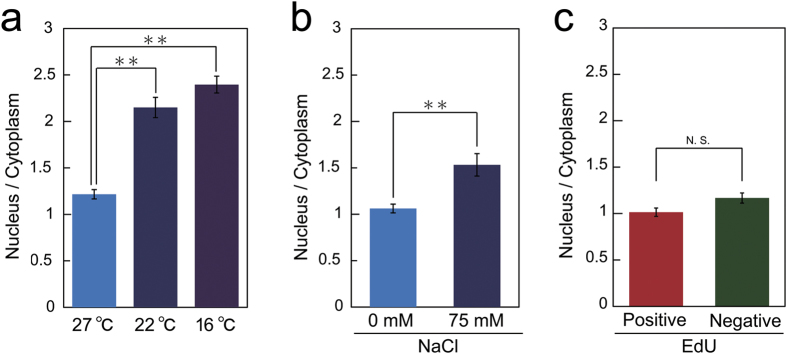
Monitoring of H3K9ac in response to environmental stresses. (**a**,**b**) Quantitative analysis of H3K9ac levels in tobacco BY-2 cells treated with cold stress for 3 h (**a**) or NaCl for 6 h (**b**). The ratio of nuclear/cytoplasmic intensity of H3K9ac-mintbody-GFP is shown. The values represent means ± S.D. (n = 16–19, *t*-test; **p < 0.01). (**c**) Quantitative analysis of H3K9ac levels during the cell cycle in tobacco BY-2 cells. Cell cycle stages were distinguished by the pulse EdU incorporation. The ratio of nuclear/cytoplasmic intensity of H3K9ac-mintbody-GFP is shown. The values represent means ± S.D. (n = 16–28, N.S., not significant by *t*-test).

Finally, we assumed that H3K9ac was also responsive to environmental stresses. The responses of H3K9ac-mintbody-GFP dynamics to environmental stresses during interphase were analysed. The nuclear/cytoplasmic intensity of H3K9ac-mintbody-GFP was increased by both cold and salt treatments (Fig. 4a,b). However, these stresses can affect cell cycle progression and the changes in the nuclear/cytoplasmic intensity of H3K9ac-mintbody-GFP might simply have reflected differences in H3K9ac levels among the cell cycle stages G1, S, and G2. To investigate this possibility, we analysed the dynamics of H3K9ac-mintbody-GFP during each cell cycle stage. The cell cycle stages were distinguished by the pulse 5-ethynyl-2′-deoxyuridine (EdU) incorporation method^31^. However, we found no significant difference in the nuclear/cytoplasmic intensity of H3K9ac-mintbody-GFP between EdU-positive (S phase) and -negative cells (G1 or G2 phase) (Fig. 4c), suggesting that H3K9ac levels may not differ with cell cycle progression during interphase. Taken together, these results suggested that both cold and salt stresses induced hyperacetylation of H3K9 in tobacco BY-2 cells, which was not attributable to the inhibitory effects of these stresses on cell cycle progression.

## Discussion

Here we established that H3K9ac-mintbody-GFP can be used as a functional tool for monitoring the acetylation status of H3K9 in cultured living plant cells. The mouse ScFV based on H3K9ac-mintbody-GFP functionally worked in plant cells without the immune system as previously reported in cultured animal cells^11^. Using this system, we succeeded in analysing a time-course of changes in H3K9 acetylation levels in response to environmental stresses and during cell cycle progression (Fig. 4). Interestingly, quantitative analysis of H3K9ac-mintbody-GFP revealed increased acetylation even during treatment with Ky-14, which has quite low inhibitory activity against HDACs (Fig. 3e,f), suggesting that H3K9ac-mintbody-GFP expressing tobacco BY-2 cells could be a powerful tool for screening novel HDAC-inhibitory chemicals.

During DNA replication, histones are acetylated and then deacetylated when the cells migrate to G2 phase. This event is highly conserved among a variety of organisms, including animals and plants^32^. However, differences in acetylation dynamics among histones H3 and H4 have been found in plants. Acetylation of H4 is linked to DNA replication, whereas H3 acetylation remains fairly constant throughout the cell cycle in field bean^33^. In the root apical meristem of *Arabidopsis thaliana*, histone mobility, which is dramatically affected by histone acetylation status, does not change during cell cycle progression^34^, suggesting constant histone acetylation levels. In line with these results, our data confirmed that the acetylation status of histone H3 at K9 remains constant irrespective of cell cycle stage in proliferating plant cells (Fig. 4c).

In our characterization of H3K9ac-mintbody-GFP, we found sustained acetylation of H3K9 throughout the mitotic phase in tobacco BY-2 cells (Fig. 2a,b). This is not consistent with a previous report in tobacco protoplast cells in which acetylation of H4 and H3K9/K14 during mitosis was undetectable^35^. However, it is possible that this difference in the dynamics of acetylation status results from differences in cellular properties. In contrast to tobacco protoplast cells, certain levels of histone H4 acetylation are maintained throughout the mitotic phase in barley^36^. In cultured human cells, histone acetylation at various residues of H3 including K9 and H4 is decreased in association with chromatin compaction^37^,^38^. Taking these results together, it is tempting to speculate that the acetylation status of histones during mitotic progression differs with cellular identity or species.

In tobacco BY-2 cells, immunoblotting analyses showed that cold and salt stresses induced an increase in acetylation of histone H4^39^. Here, H3K9ac-mintbody-GFP showed enhanced H3K9ac in tobacco BY-2 cells under cold and salt stresses (Fig. 4a,b). Together, these results suggest that global histone acetylation occurs in tobacco BY-2 cells under these stresses. The mintbody system is a useful system to analyse the timing of genome-wide histone modification changes after environmental stimuli. Similarly, global changes of H3K9ac in response to drought, light, and DNA damage have been observed in *A. thaliana*^19^,^20^,^21^,^22^,^23^,^24^. Therefore, it is also worth monitoring H3K9ac responses to environmental stimuli in plants. However, at this moment, we have not succeeded in generating plants expressing functional H3K9ac-mintbody-GFP for quantitative analysis as H3K9ac-mintbody-GFP was only detected in nuclei under the control of the CaMV 35S or RPS5a promoter (Supplementary Fig. S3). This may be due to the non-functional folding of H3K9ac-mintbody-GFP or the preferential localization of H3K9ac-mintbody-GFP to nucleus in *A. thaliana*. In the former case, it would be worth trying the different clone of scFV for H3K9ac a few with a few different amino acids^11^. In the latter case, we could use a nuclear exporting signal to obligatorily express H3K9ac-mintbody-GFP in both the cytoplasm and nucleus.

Because plant organs and tissues comprise various types of cells such as epidermal and endodermal cells, each cell type might have specific epigenetic regulation upon environmental and developmental stimuli. Recently, the DNA methylation status, an epigenetic mark, has been directly shown to differ among cell types in roots using a cell-sorting technique^40^. Regarding histone modification, although the acetylation levels of histone H4 have been shown to differ between dividing and differentiated cells in roots^34^, to our knowledge, there have been no reports on cell type-specific histone modification status. Using a mintbody, we could detect differences in modification status among cell types in living plants. In addition, our data demonstrating that H3K9ac-mintbody-GFP is a functional endogenous antibody in tobacco BY-2 cells (Fig. 1b,c and Supplementary Fig. S1) indicate that a mintbody could be used for cell type-specific ChIP if expressed under a cell type-specific promoter. The application of mintbodies for various types of modifications such as H3K27me3^11^ and H4K20me1^14^ in addition to H3K9ac will help us to understand the orchestrated epigenetic programs specific to different cell types.

## Methods

### Cell materials and culture conditions

Tobacco BY-2 cells were grown in MS (Murashige and Skoog) medium containing MS salts, 3% sucrose, 200 mg/L KH_2_PO_4_, 100 mg/L myo-inositol, 1 mg/L thiamine HCl and 0.2 mg/L 2,4-D, at pH 5.8 with KOH, at 27 °C on a shaking platform operating at 130 rpm in the dark. The cells were subcultured by 100-fold dilution in fresh medium every 7 days.

Tobacco BY-2 cells expressing CaMV 2 × pro35S::19E5scFv-GFP (H3K9ac-mintoboy-GFP) were constructed using the following procedures. The DNA sequence encoding 19E5scFv-GFP^11^ was amplified by PCR and subcloned into the pENTR/D-TOPO vector (Thermo Fisher Scientific, http://www.thermofisher.co.jp/), and then recombined into the pMDC32 binary vector using LR Clonase II (Thermo Fisher Scientific). The PCR primers used were 5′-CACCATGGCCGAGGTCCAGCTGCAGC-3′ and 5′-TTACTTGTACAGCTCGTCCATG-3′. Then, the constructed binary vector was introduced into *Agrobacterium tumefaciens* (EHA105) and used to transform tobacco BY-2 cells as previously described^41^. To generate transgenic *A. thaliana* expressing 2 × pro35S::19E5scFv-GFP and proRPS5a::19E5scFv-GFP, the same binary vector for tobacco BY-2 cells and pGWB501 containing proRPS5a binary vector^42^ was used, respectively.

### Immunofluorescence staining of BY-2 cells

Slide preparation and immunofluorescence staining procedures were carried out as described previously^43^. Four-day-old subcultured BY-2 cells were used to prepare slides. To detect H3K9ac and microtubules, anti-H3K9ac (ab4441, Abcam, http://www.abcam.co.jp/) and anti-α-tubulin (DM1A, CP06, Merck Millipore, http://www.merckmillipore.com) were used at 1:1,000 dilutions. To visualize nuclei and chromosomes, specimens were mounted with mounting medium (100 mM Tris, 50% glycerol, 1 mg/mL *o*-phenylene diamine, pH 9.2) containing DAPI (1 μg/mL, Roche, http://www.roche.com/). Image acquisition was performed using a Olympus FV1200 confocal microscope (Olympus, http://www.olympus-global.com/).

### Chemical and stress treatments

Different concentrations of TSA (Wako, http://www.wako-chem.co.jp/), C646 (Sigma, http://www.sigmaaldrich.com/japan.html/), Ky-2, Ky-14 or NaCl (Wako) were added to medium containing tobacco BY-2 cells 3 days after subculture. Cold stress treatment was performed by transferring cells 3 days after subculture to an incubator adjusted to 22 °C or 16 °C.

### EdU incorporation assay

The EdU incorporation assay was carried out as described previously^44^. EdU was detected with the Click-iT Plus EdU Alexa Fluor 594 Imaging Kit (Thermo Fisher Scientific) following the manufacturer’s guidelines. For EdU incorporation, tobacco BY-2 cells subcultured after 3 days were treated with 10 μM EdU for 30 min. The cells were fixed for 20 min in 4% (w/v) paraformaldehyde in PBS (pH 7.4), washed twice with PBS and placed in 0.5% (v/v) TritonX-100 in PBS. After 20 min, the samples were washed with PBS twice and incubated in the Click-iT reaction cocktail for 30 min in the dark. The Click-iT reaction cocktail was removed and the samples were washed with PBS once.

### Imaging systems and analysis

For the time-lapse imaging, the samples were observed under an FV1200 inverted laser confocal microscope equipped with a GaAsP detector (Olympus, http://www.olympus-lifescience.com/ja/) using a 473 nm laser for GFP. To quantify the H3K9ac-mintbody-GFP intensity, the samples were observed under an inverted fluorescence microscope (IX81, Olympus), which included a laser (488 nm for GFP and 561 nm for Alexa Fluor 594 for EdU detection), equipped with a confocal scanning unit (CSU-X1, Yokogawa, http://www.yokogawa.co.jp/) and a sCMOS camera (Neo 5.5 sCMOS, ANDOR, http://www.andor.com/). The images were analysed with the Image J software (US National Institutes of Health, Bethesda, MD, USA, http://imagej.nih.gov/ij/). The images are shown in 16 colours; cold and warm colours indicate lower and higher GFP intensities, respectively.

### Immunoprecipitation

Tobacco BY-2 cells 3 days after subculture were treated with 1 μM TSA or 10 μM C646 for 3 h. The cells were gathered by centrifugation at 800 rpm for 1 min and then homogenized by grinding with a mortar and a pestle in liquid nitrogen. Next, 500 μL of lysis buffer (25 mM HEPES pH 7.5, 150 mM NaCl, 1 mM EDTA, 1% TritonX-100, 0.1% Na-deoxycholate, 0.1% SDS, 5 mM sodium butyrate and a protease inhibitor cocktail (P9599, Sigma)) was added to the ground powder. The extract was filtered through two layers of Miracloth (Merck Millipore).

Immunoprecipitation was performed with Dynabeads M-280 coated with sheep anti-rabbit IgG (Thermo Fisher Scientific) following the manufacturer’s guideline. First, 1 μg anti-acetyl-histone H3 (Lys9) antibody (ab4441, Abcam, http://www.abcam.co.jp/) or 2 μg anti-dimethyl-histone H3 (Lys9) antibody (07-441, Merck Millipore, https://www.merckmillipore.com/) was added to 50 μL of pre-washed Dynabeads M-280. To couple the beads with the target Ig, the mixture was incubated for 1 h at 4 °C with gentle rotation. Then, the target Ig-coupled beads were co-incubated with the cell extract for 2 h at 4 °C with gentle rotation. The immunoprecipitated proteins were eluted from the beads by incubating with SDS sample buffer for 15 min at 95 °C. The primary antibodies used for the immunoblotting of GFP, H3K9ac, H3K9me2, and H3 were anti-GFP (11814460001, Roche, https://roche-biochem.jp/), anti-acetyl-histone H3 (Lys9) (ab4441, Abcam), anti-dimethyl-histone H3 (Lys9) (07-441, Merck Millipore) and anti-histone H3 (MA301, MBL, http://www.mbl.co.jp/), respectively. The secondary antibodies were HRP-conjugated anti-mouse IgG (W402, Promega, https://www.promega.jp/) or anti-rabbit IgG (MB4458, MBL). A Fusion Pulse (Vilber Lourmat, http://www.vilber.de/en/home/) was used for the detection of chemiluminescence.

### Nuclear isolation

Tobacco BY-2 cells 3 days after subculture were treated with 1 μM TSA or 10 μM C646 for 3 h. The cells were collected by centrifugation at 800 rpm for 1 min. Nuclear isolation from BY-2 cells was carried out as described previously^45^.

### Synthesis of Ky-14

To a solution of cyclo(-L-2-amino-8-oxo-9-bromononanoyl-aminoisobutylyl-L-phenylalanyl-D-proryl-) (63 mg) in anhydrous methanol (0.5 mL) 2 M dimethylamine/methanol (0.082 mL) was added under stirring. After 5 h, the desired cyclic tetrapeptide was isolated into ethyl acetate. The crude product was purified by silica gel chromatography with a mixture of chloroform and methanol (95/5, v/v). Yield, 40 mg, 68%). HPLC, rt 4.54 min. HR-FABMS [M + H]^+^ 542.3320 for C_29_H_44_O_5_N_5_ (calcd. 542.3342). ^1^H-NMR (500 MHz, CDCl_3_: δ_H_ 1.31 (2 H, m, CHCH_2_CH_2_CH_2_), 1.32 (2 H, m, CHCH_2_CH_2_), 1.32 and 1.59 (1 H each, each, m, (2 H, m, CHCH_2_CH_2_CH_2_CH_2_), 1.34 (3 H, s, CH_3_), 1.58 and 1.79 (1 H each, each, m, CHCH_2_), 1.74 and 2.32 (1 H each, each, m, CHCH_2_), 1.79 and 2.17 (1 H each, each, m, CHCH_2_CH_2_), 1.77 (3 H, s, CH_3_), 2.29 (6 H, s, CH_2_COCH_2_N(CH_3_)_2_), 2.42 (2 H, t, J = 7.5 Hz CH_2_COCH_2_N(CH_3_)_2_) 2.95 (1 H, dd, J = 13.5, 6.0 Hz CHCH_2_), 3.14 (2 H, s, CH_2_COCH_2_N(CH_3_)_2_), 3.21 and 3.86 (1 H each, each, m, CHCH_2_CH_2_CH_2_), 3.26 (1 H, ddd, J = 10.2, 10,2, 5.5 Hz, CH), 5.94 (1 H, s, NH), 7.09 (1 H, d, J = 10.5 Hz, NH), 7.21 (3 H, m), 7.27 (2 H, m), 7.50 (1 H, d, J = 10.5 Hz, NH).

## Additional Information

**How to cite this article**: Kurita, K. *et al*. Live imaging of H3K9 acetylation in plant cells. *Sci. Rep.* **7**, 45894; doi: 10.1038/srep45894 (2017).

**Publisher's note:** Springer Nature remains neutral with regard to jurisdictional claims in published maps and institutional affiliations.

## Supplementary Material

Supplementary Information
