# Supplementary material for: Live imaging of H3K9 acetylation in plant cells
Source: Sci Rep. 2017 Apr 18;7:45894. doi: 10.1038/srep45894 (PMC5394682; doi:10.1038/srep45894)
Supplement: Supplementary Information [file srep45894-s1.pdf]

Supplementary information

Live imaging of H3K9 acetylation in plant cells

Kazuki Kurita[1,7], Takuya Sakamoto[1,7] , Noriyoshi Yagi[1], Yuki Sakamoto[2], Akihiro Ito[3], Norikazu Nishino[4], Kaori Sako[5], Minoru Yoshida[3], Hiroshi Kimura[6], Motoaki Seki[5], and Sachihiro Matsunaga[1,2*]

[1]*Department of Applied Biological Science, Faculty of Science and Technology, Tokyo University of Science, 2641 Yamazaki, Noda, Chiba 278-8510 Japan*

[2]*Imaging Frontier Center, Organization for Research Advancement, Tokyo University of Science, 2641 Yamazaki, Noda, Chiba 278-8510 Japan*

[3]*Chemical Genomics Research Group, RIKEN Center for Sustainable Resource Science, 2-1 Hirosawa, Wako, Saitama, 351-0198 Japan*

[4]*Graduate School of Life Science and Systems Engineering, Kyushu Institute of Technology, Kitakyushu, 808-0196 Japan*

[5]*Plant Genomic Network Research Team, RIKEN Center for Sustainable Resource Science, 1-7-22 Suehiro-cho, Tsurumi-ku, Yokohama, Kanagawa, 230-0045 Japan*

[6]*Graduate School of Bioscience and Biotechnology, Tokyo Institute of Technology, 4259 Nagatsuta, Midori-ku, Yokohama, 226-8501 Japan*

[7]These authors equally contributed to this work.

*For correspondence

Sachihiro Matsunaga

Department of Applied Biological Science, Faculty of Science and Technology, Tokyo University of Science, 2641 Yamazaki, Noda, Chiba 278-8510, Japan.

Phone: +81-4-7124-1501 Fax: +81-4-7122-9104 E-mail: sachi@rs.tus.ac.jp

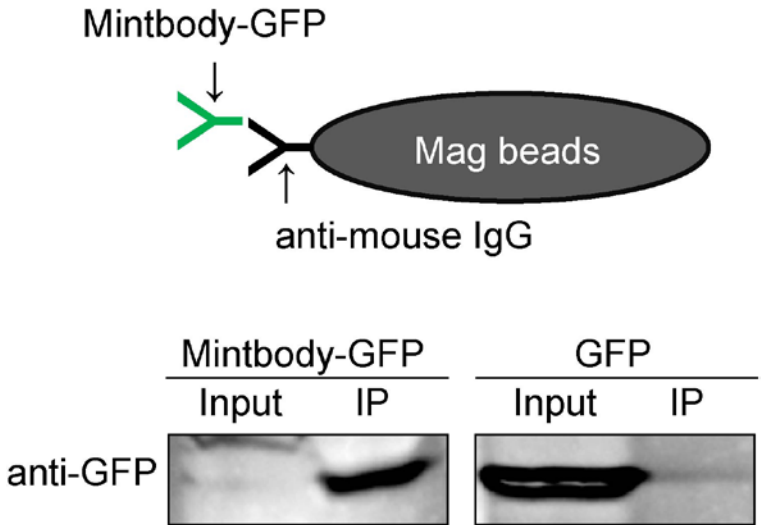

Supplementary Figure S1. H3K9ac-mintbody-GFP expressed in plant cells can bind to anti-mouse IgG.

Whole cell extracts from H3K9ac-mintbody-GFP or GFP expressing tobacco BY-2 cells were subjected to immunoprecipitation with anti-mouse IgG antibody on magnetic beads. After the immunoprecipitation, H3K9ac-mintbody-GFP and GFP were detected by immunoblotting. Only H3K9ac-mintbody-GFP was immunoprecipitated with the anti-mouse IgG antibody. For the full-length western blots see Supplementary Fig. S5.

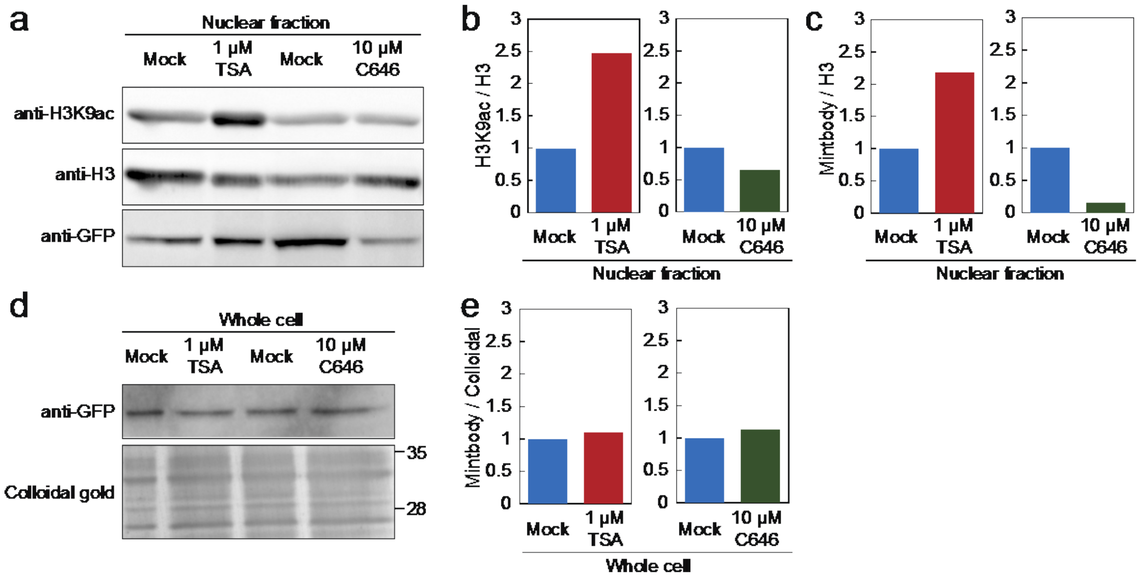

Supplementary Figure S2. The amount of nuclear H3K9ac-mintbody-GFP was correlated with the amount of H3K9ac in tobacco BY-2 cells.

(a) Immunoblotting analysis of H3K9ac, H3, and H3K9ac-mintbody-GFP in the nuclear fractions of tobacco BY-2 cells treated with 1 μM TSA and 10 μM C646. The H3 level was used as a loading control. For the full-length western blots see Supplementary Fig. S6a.

(b, c) Quantitative analysis of the levels of nuclear H3K9ac (b) and H3K9ac-mintbody-GFP (c). The intensity of each band shown in (a) was analysed with ImageJ. Each protein level was normalized to the H3 level.

(d) Immunoblotting analysis of H3K9ac-mintbody-GFP in whole cell fractions of tobacco BY-2 cells treated with 1 μM TSA and 10 μM C646. The loaded proteins were stained with colloidal gold. For the full-length western blots see Supplementary Fig. S6b.

(e) Quantitative analysis of the levels of whole cell H3K9ac-mintbody-GFP. The intensity of each band shown in (d) was analysed with ImageJ. Each protein level was normalized to the level of the proteins stained with colloidal gold.

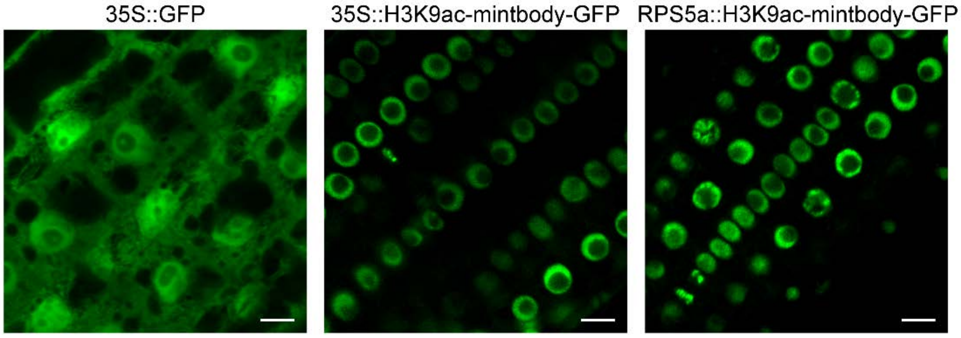

Supplementary Figure S3. H3K9ac-mintbody-GFP is unlikely to be expressed in the cytoplasm under the control of the 35S or RPS5a promoter.

Root cells of *A. thaliana* expressing GFP and H3K9ac-mintbody-GFP under the control of the 35S or RPS5a promoter were observed by confocal laser microscopy. T_2 plants were used for the H3K9ac-mintbody-GFP observations. Compared with GFP alone, H3K9ac-mintbody-GFP was not visible in the cytoplasm. Bars, 10 µm.

Supplementary Table S1. Characteristics of Ky-2 and Ky-14.

	R	composition	Mw	*in vitro* IC50 inhibitory activity (±S.D.)		
				HDAC1	HDAC4	HDAC6
Ky-2	CONHOH	$C_{26}H_{37}O_6N_5$	515.60	0.00873 μM ± 0.00056	0.01997 μM ± 0.00217	0.16345 μM ± 0.01243
Ky-14	$COCH_2N(CH_3)_3$	$C_{29}H_{32}O_5N_5$	541.68	19.98 μM ± 3.16	17.86 μM ± 1.93	>100 μM

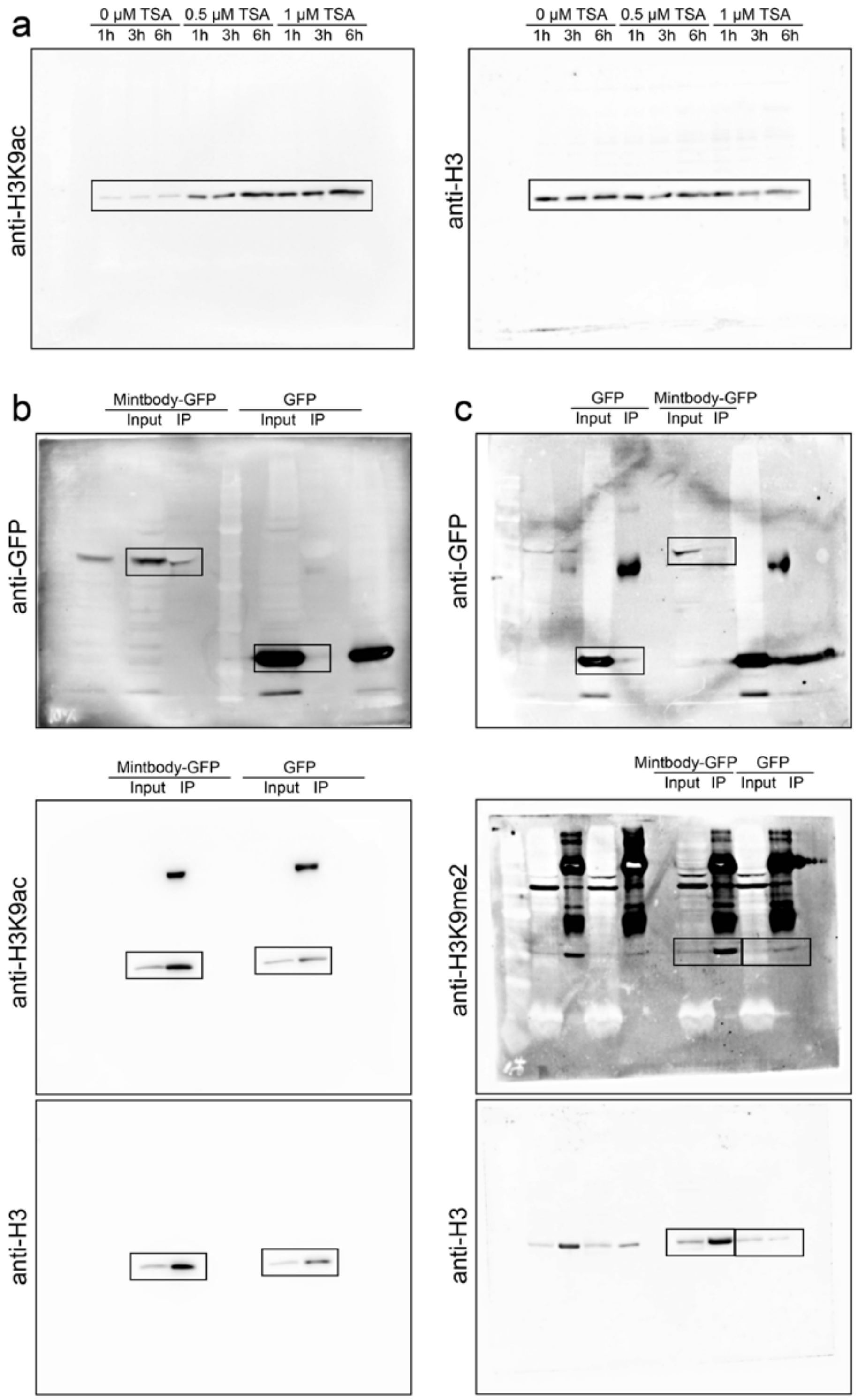

Supplementary Figure S4. Full-length Western blots from Fig.1.

Full-length western blot shown in Figure 1a (a), in Figure 1b (b), and in Figure 1c (c). Lanes used are outline with a black rectangle.

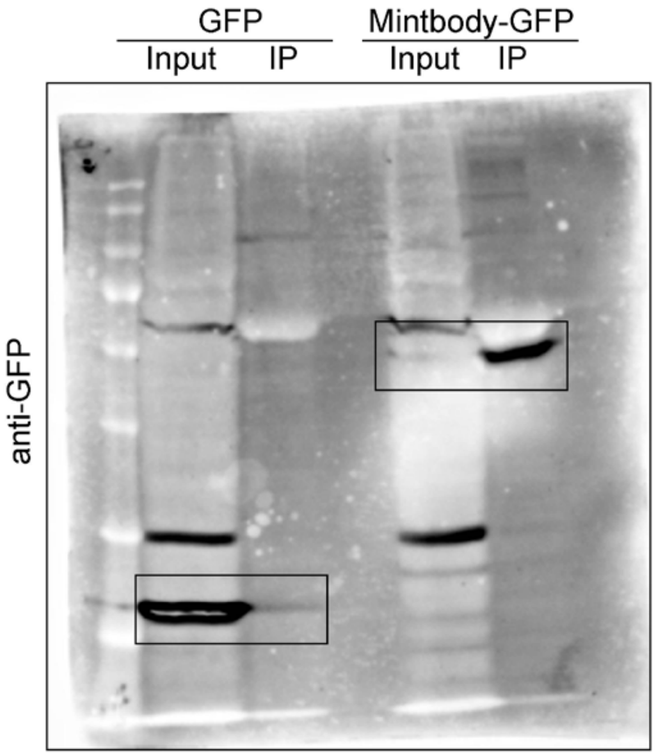

Supplementary Figure S5. Full-length Western blots from Supplementary Fig.S1.

Lanes used are outline with a black rectangle.

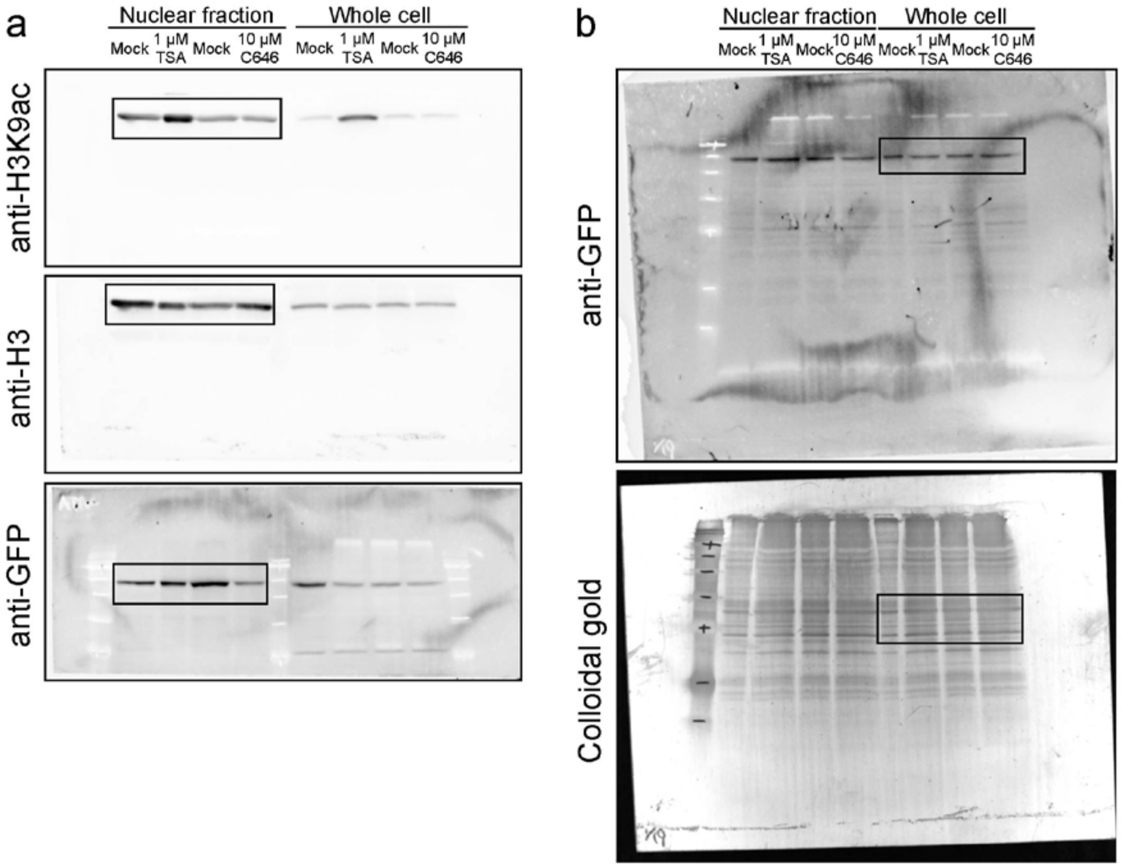

Supplementary Figure S6. Full-length Western blots from Supplementary Fig.S2.

Full-length western blot shown in Supplementary Figure S2a (a), and in Supplementary Figure S2d (b). Lanes used are outline with a black rectangle.
